# Pulsed Field Ablation of the Porcine Ventricle Using a Focal Lattice-Tip Catheter

**DOI:** 10.1161/CIRCEP.122.011120

**Published:** 2022-09-08

**Authors:** Iwanari Kawamura, Vivek Y. Reddy, Bingyan J. Wang, Srinivas R. Dukkipati, Hina W. Chaudhry, Carlos G Santos-Gallego, Jacob S. Koruth

**Affiliations:** 1 Helmsley Electrophysiology Center (I.K., V.Y.R., S.R.D., J.S.K.), Icahn School of Medicine at Mount Sinai, New York, NY.; 2 Cardiovascular Regenerative Medicine (B.J.W., H.W.C.), Icahn School of Medicine at Mount Sinai, New York, NY.; 3 Atherothrombosis Research Unit, Department of Cardiology (C.G.S.-G.), Icahn School of Medicine at Mount Sinai, New York, NY.

**Keywords:** catheter ablation, electroporation, immunohistochemistry, myocardium, swine, tachycardia,ventricular

## Abstract

Our understanding of catheter-based pulsed field ablation (PFA) of the ventricular myocardium is limited. We conducted a series of exploratory evaluations of ventricular PFA in swine ventricles.

**Methods:**

A focal lattice-tip catheter was used to deliver proprietary biphasic monopolar PFA applications to swine ventricles under general anesthesia, with guidance from electroanatomical mapping, fluoroscopy, and intracardiac echocardiography. We conducted experiments to assess the impact of (1) delivery repetition (2×, 3×, or 4×) at each location, (2) epicardial PFA delivery, and (3) confluent areas of shallow healed endocardial scar created by prior PFA (4 weeks earlier) on subsequent endocardial PFA. Additional assessments included PFA optimized for the ventricle, lesion visualization by intracardiac echocardiography imaging, and immunohistochemical insights.

**Results:**

Experiment no. 1: lesions (n=49) were larger with delivery repetition of either 4× or 3× versus 2×: length 17.6±3.9 or 14.2±2.0 versus 12.7±2.0 mm (*P*<0.01, *P*=0.22), width 13.4±1.8 or 10.6±1.3 versus 10.5±1.1 mm (*P*<0.01, *P*=1.00), and depth 6.1±2.1 or 5.1±1.3 versus 4.2±1.0 mm (*P*<0.01, *P*=0.21). Experiment no. 2: epicardial lesions (n=18) were reliably created and comparable to endocardial lesions: length 24.6±9.7 mm (n=5), width 15.6±4.6 mm, and depth 4.5±3.7 mm. Experiment no. 3: PFA (n=16) was able to penetrate to a depth of 4.8 (interquartile range, 4.5–5.4) mm in healthy myocardium versus 5.6 (interquartile range, 3.6–6.6) mm in adjacent healed endocardial scar (*P*=0.79), suggesting that superficial scar does not significantly impair PFA. Finally, we demonstrate, PFA optimized for the ventricle yielded adequate lesion dimensions, can result in myocardial activation, can be visualized by intracardiac echocardiography, and have unique immunohistochemical characteristics.

**Conclusions:**

This in vivo evaluation offers insights into the behavior of endocardial or epicardial PFA delivered using the lattice-tip catheter to normal or scarred porcine ventricular myocardium, thereby setting the stage for future clinical studies.

**What is Known?**

- Catheter-based pulsed field ablation has been explored as a novel energy source for cardiac ablation.
- There is a need to elaborate on the effect of catheter-based pulsed field ablation on ventricular myocardium.

**What the Study Adds**

- Endocardial ventricular lesions are circular/oval in shape consistent with the expected geometry of the electrical field and repetitive PF applications (2 to 4) progressively improve lesion depth —a mean depth of 6 mm, with length and width of ≈1.5 cm, is achievable.
- Epicardial pulsed field ablation is feasible and creates lesions, penetrating ~4 mm epicardial fat, with comparable dimensions, with preservation of surface contours.
- Endocardial scarring up to 4 mm depth did not impair subsequent pulsed field ablation from penetrating the myocardium beyond the scar.

Catheter-based pulsed field ablation (PFA) has been explored as a novel energy source for cardiac ablation, with several reports demonstrating promising data.^1-8^ PFA’s nonthermal properties and ability to spare collateral organs, such as the esophagus, makes it unique amongst contemporary ablative energy sources. Although well studied in both preclinical and clinical studies for its ability to ablate atrial tissue, PFA’s role in the ventricle remains unclear. In theory, catheters designed to deliver focal PFA lesions have the potential to become an attractive alternative to currently available radiofrequency catheters for ventricular ablation, especially if they can deliver deep lesions consistently and safely.^4-8^ We previously described focal PFA delivery in swine ventricles using a 12F multielectrode, focal endocardial catheter with lesions as deep as 6.5±1.7 mm. In addition, we have described focal PFA applications using a 7.5F focal lattice-tip catheter that achieve depths of 2.9±1.0 mm as part of initial atrial ablation dosing studies.^5^ This lattice-tip catheter seems well-suited for ablation of ventricular arrhythmias (VAs), particularly as it is linked to an electroanatomical mapping system. Indeed, recent studies have expanded the ability of this lattice-tip catheter to generate ventricular lesions.^7^ Accordingly, in this preclinical series of porcine experiments, we explored focal ventricular PFA using the lattice-tip catheter to assess: the impact of PF delivery repetition, the effect of PFA on healed endocardial scar, epicardial ventricular PFA, the effect of PFA delivery optimized for ventricular tissue, ultrasonic lesion visualization, and immunohistochemical staining characteristics.

## Methods

**Figure 1.**
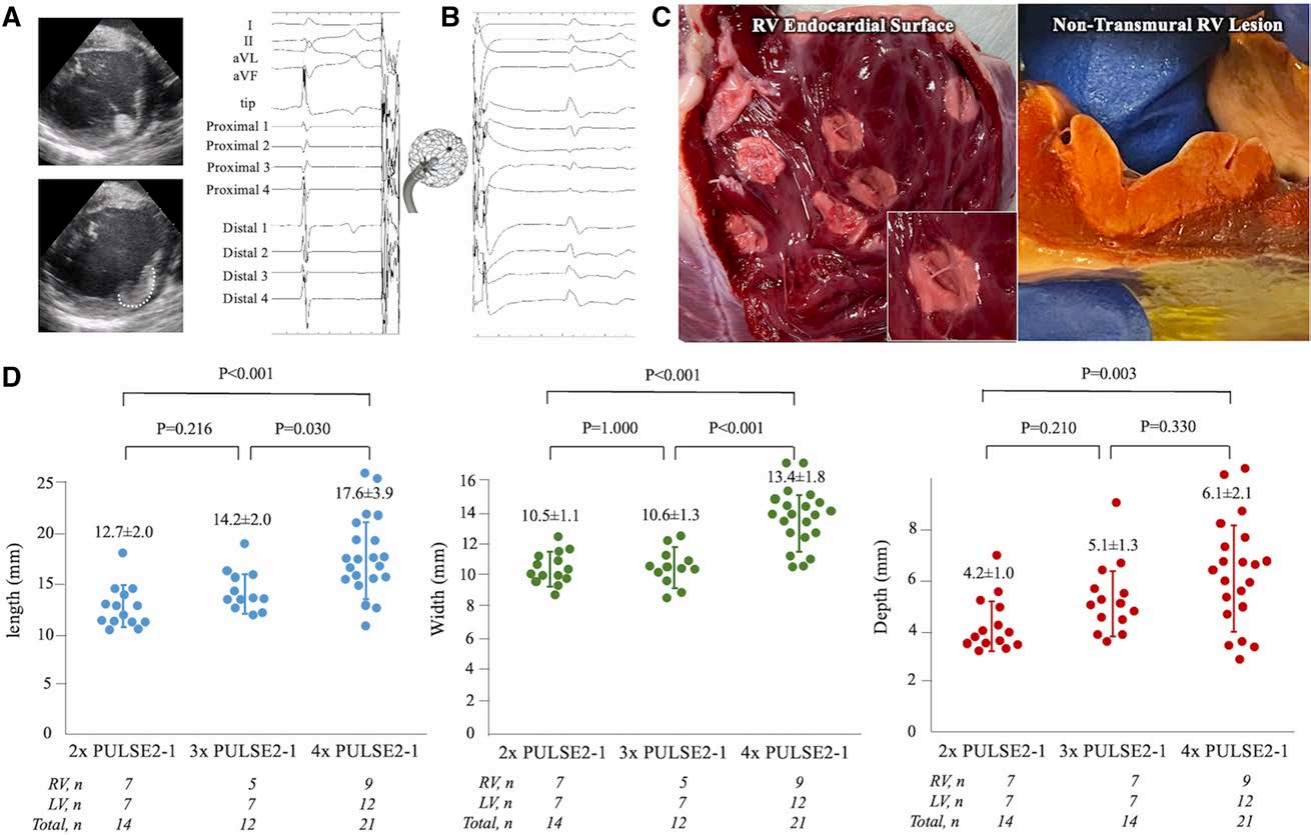
**Endocardial pulsed field ablation lesions. A**, Lattice-tip catheter placed against the left ventricular (LV) wall (**upper**) with outline of lesion observed minutes later on intracardiac echocardiography (**lower**), **B**, Microelectrode electrograms before ablation (**left**) and after ablation (**right**). **C**. Triphenyl tetrazolium chloride stained ventricle with discrete endocardial lesions demonstrating clear, oval, homogenous ablations (**left**) and a lesion demonstrating near transmural extent (**right**). **D**, Length, width, and depth of lesions created by 2, 3, and 4 PULSE2-1 applications per site. RV indicates right ventricle.

Data and methods used in the analysis and materials used to conduct the research will not be available for access. All preclinical experiments were approved by the Institutional Animal Care and Use Committee at the Mount Sinai Hospital, New York. Female Yorkshire swine (60–70 kg) were included for this evaluation. Under general anesthesia and via transfemoral venous access, the study catheter was introduced into the right and left ventricles (RVs and LVs) using a deflectable sheath (Agilis, Abbott, Inc). Transeptal access was used for endocardial ablation of the LV and standard subxyphoid percutaneous epicardial access for epicardial ablation. The study catheter has a lattice-tip (Sphere-9; Affera, Inc, Watertown, MA) and is capable of both mapping (Prism-1; Affera, Inc) as well as ablation via a generator capable of delivering PFA (HexaPULSE; Affera, Inc). As previously described, this 7.5F bidirectionally-deflectable catheter has a tip containing 9 mini-electrodes/temperature sensors and location sensors allowing for 3-dimensional localization.^4,5,7^ PFA is applied as a monopolar field from the entire lattice tip (to a grounding electrode) using a proprietary biphasic waveform. The catheter is irrigated at 4 and 15 mL/min at baseline and during ablation, respectively. An activated clotting time of 300 to 400 s was targeted. The catheter tip was monitored using intracardiac echocardiography (ICE)/electroanatomic mapping to ensure stability during applications (Figure 1A). The largest bipolar voltage recorded from the 9 surface microelectrodes was identified and recorded before and immediately after PFA (Figure 1B).

A series of experiments were focused on PFA application in swine ventricles. Dosing decisions differed between experiments, and these were made based on serial, evolutionary evaluations.

### Experiments

#### Experiment No. 1: Endocardial PFA Repetition Study

Experimental PFA pulses of 1-s duration (±2 kV, PULSE2-1) were applied in both ventricles in 4 healthy swine. We assessed the impact of three dosing strategies (variable repetition) on lesion dimensions: 1-s duration pulses were repeated 2×, 3×, or 4× at each location. Repetitions were performed at 10 to 15 s intervals. After surviving swine for 24 hours, they were sacrificed humanely for further analysis.

#### Experiment No. 2: Epicardial PFA Study

PFA was delivered in both ventricles in 3 healthy swine. We elected to deliver PFA pulses with a 5.5-s setting (±2 kV, PULSE3V) optimized for ventricular delivery based on prior optimal dosing studies (above as well as others) to maximize lesion size with a single delivery. Subxiphoid access was obtained in 2 swine, and epicardial lesions were delivered in discrete locations using a deflectable sheath. The remaining 1 swine received only endocardial applications because of failure to obtain percutaneous epicardial access. All animals were survived for ≈48 hours and then humanely killed for analysis.

#### Experiment No. 3: Endocardial PFA over Healed Scar Study

Two to 4 PFA lesion clusters were created, using a 4-s setting (±2 kV, PULSE3) originally optimized for atrial delivery, in both ventricles (endocardium) in four healthy swine. All animals were then survived for 4 weeks to create wide confluent areas of shallow endocardial scarring. Instead of a traditional infarct-scar model, we used this scar model to test the interaction of PFA with scar as this model provided homogenous and easy-to-identify scar. In the second study after 4 weeks, scar was identified as low voltage regions on the electroanatomical map (created using the lattice-tip catheter) and additional acute PFA lesions were applied at the border of the scar, such that lesions overlapped both scarred and heathy myocardium (Figure S1). The objective was to examine if endocardial scar impaired the depth of PFA penetration by comparing acute PFA lesion depth immediately below scarred regions versus that in the neighboring normal myocardium. All animals were survived for 24 hours after the second procedure and then humanely sacrificed for analysis.

### Lesion Evaluation

Triphenyl tetrazolium chloride staining was performed to identify PFA applications and used for all experiments. The chest was opened, and the heart, pericardium, and lungs were surveyed and inspected. We did not attempt to correlate lesions to those recorded on the electroanatomic map given that dosing strategies were never changed within chambers. The surface width and length were first measured before sectioning. Lesions were then sectioned in the midline along the long axis and the depth was recorded. All tissues were fixed in formalin solution for histological analysis in select specimens. Hematoxylin and eosin and Masson Trichrome staining were performed and were interpreted by a veterinary pathologist.

### Immunohistochemistry

A subset of select lesions underwent immunohistochemical staining in 1 swine. Formalin-fixed paraffin-embedded heart sections were deparaffinized by serial immersion of Xylene and Absolute, 95%, 70% Ethanol. Autofluorescence background was quenched in 0.25% Sudan Black B (S-2380, in 70% ethanol; Sigma-Aldrich) for 5 minutes at room temperature. Antigen retrieval was achieved by microwaving slides in Citrus Buffer (pH 6.0) immersion at near boiling point for 15 minutes. Endogenous peroxidase activity was quenched by incubating in 3% H_2_O_2_ for 20 minutes at room temperature. Slides were then blocked with 10% horse serum (26050088; Gibco) and incubated in primary antibodies (1:100) at 4 ºC overnight. The next day, slides were washed in 1× TBST (BP24711; Fisher) with 0.05% Tween-20 and incubated in secondary antibodies (1:200) for 60 minutes at room temperature. Antibodies used included: cTnI (cardiac Troponin I, ab56357; Abcam), cTnT (cardiac Troponin T, 701620; ThermoFisher), Col IV (collagen IV, AB769; Millipore), CX43 (connexin-43, 13-8300; ThermoFisher). CX43 protein staining was amplified using anti-mouse horseradish peroxidse (SA1-100; Invitrogen) and fluorescein isothiocyanate (FITC) tyramide signal amplification fluorescence system kit (K1050; APExBIO). Cell membranes were stained with WGA (wheat germ agglutinin) 680 conjugate (W32465, 10 µg/mL; ThermoFisher) for 60 minutes at 37 °C. Nuclei were counterstained with DAPI (0.1 µg/mL; ThermoFisher 62247) for 15 minutes at room temperature. Slides were then mounted in Mounting media (KPL 71-00-16) before imaging by Leica SP8 confocal microscopy and Leica LAS X analysis software. Representative images are from 10 to 20 scans of each zone (remote, border, lesion) from 6 different sections.

### Statistical Analysis

Continuous variables are expressed as mean±SD or median with interquartile range, and categorical variables are given as count and percentage. Continuous variables were compared between the groups via the Student *t* test or Mann-Whitney *U* test, and categorical variables were compared by χ2 analysis or Fisher test, as appropriate. To compare paired data (such as bipolar voltage changes before and immediately after PFA), paired *t* test was performed. *P*<0.05 was considered significant. Bonferroni corrected *P* values were calculated to control for multiple tests of significance. Statistical analyses were performed with SPSS 24.0 software (SPSS, Inc, Chicago, IL).

## Results

### Gross Pathology

There was no gross pathological evidence of pericardial or pulmonary parenchymal trauma or appreciation of lesion extension to these and other surrounding organs. Ablation lesions were consistently identified on gross examination. The pale appearance of lesions was easily contrasted with healthy myocardium at the 24- to 48-hour time frame (Figure 1C). The lesions were predominantly circular to oval in shape, with a smooth, pale-white, homogenous appearance, and rare minimal areas of endocardial hemorrhage. The lesions had discrete margins with no transition zone visible on gross examination (Figure 1C). Localized tissue thickening extending beyond the visually apparent PFA lesions was palpable. In trabeculated regions, trabeculae were always homogenously ablated and the oval endocardial footprint was preserved and similar to smoother portions of the ventricle. The 2 swine with epicardial access demonstrated focal areas of hemorrhagic staining of the pericardium—likely related to percutaneous access attempts.

### Experiment No. 1

Discrete PFA lesions (n=50) were delivered: 24 in the RV and 26 in the LV. These applications were not optimized for ventricular delivery, but no VAs were induced by these applications despite the intracardiac echocardiography demonstrating evidence of myocardial activation during pulse delivery. The largest bipolar voltage recorded from surface microelectrodes was identified (for all lesions) and was noted to be significantly reduced from 3.11±1.19 mv (baseline) to 0.89±0.45 mv postablation (*P*<0.001), with loss of the baseline high-frequency appearance (Figure 1B). The percentage reduction of amplitude (≈67.9%) between cohorts was not significantly different despite additional repeat applications.

On gross examination, 49 of 50 PFA lesions were detected. Transmural lesions were observed in 9 of 23 (39%) RV and 1 of 26 (4%) LV lesions. Of the 9 transmural RV lesions, the distribution of application strategies was as follows for the various cohorts: 2 of 7 lesions transmural with 2-repeat applications, 3 of 7 lesions transmural with 3-repeat applications, and 4 of 9 lesions transmural with 4-repeat applications. Lesions were larger with delivery repetition of either 4× or 3× versus 2× – length 17.6±3.9 or 14.2±2.0 versus 12.7±2.0 mm (*P*<0.001, *P*=0.216), width 13.4±1.8 or 10.6±1.3 versus 10.5±1.1 mm (*P*<0.001, *P*=1.000), and depth 6.1±2.1 or 5.1±1.3 versus 4.2±1.0 mm (*P*=0.003, *P*=0.210). Furthermore, larger lesions were noted with the 4× applications in comparison to the 3× applications (length 17.6±3.9 versus 14.2±2.0 mm [*P*=0.030], width 13.4±1.8 versus 10.6±1.3 mm [*P*<0.001], and depth 6.1±2.1 versus 5.1±1.3 mm [*P*=0.330]; Figure 1D).

### Experiment No. 2

**Figure 2.**
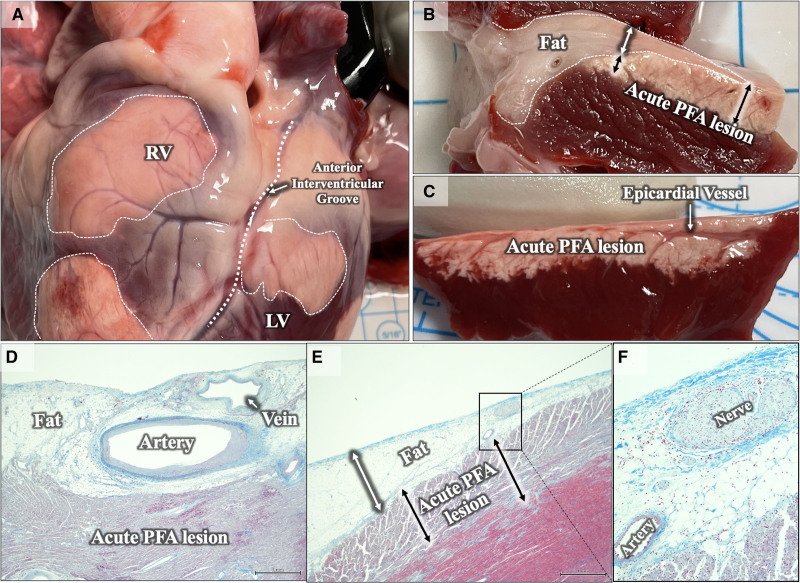
**Epicardial pulsed field ablation (PFA) lesions. A**, Epicardial lesions (dotted while outlines) over the right and left ventricles (RV and LV). Note clear preservation of vascular structures/contour on the surface of ablation lesions. **B**, Section of a PFA lesion(s) extending into an area of epicardial fat (white dotted outline). Lesion depth (black double arrows) in this region appears to be not impacted by presence of small amounts of epicardial fat (white double arrow). **C**, Section of epicardial lesion demonstrating sparing of superficial epicardial vessel and penetrating branches. **D–F**, Histology confirming epicardial lesions extending beyond myocardial fat, with sparing of veins, arteries, and nerves.

Discrete PFA lesions (n=28) were successfully delivered: 18 on the epicardium and 10 on the RV endocardium. The PULSE3V delivery was optimized for ventricular delivery as compared to the PULSE2-1 setting used in experiment 1 and the PULSE3 setting used in experiment 3. Of the 18 epicardial lesions, 13 lesions were noted to be confluent. Epicardial lesions were easily visible and had a homogenous appearance with a remarkable preservation of surface contours of the normal epicardium (Figure 2A). Lesion boundaries were discrete as with endocardial lesions and there was no evidence of lung parenchymal involvement.

The lesion length was calculated only for discrete lesions (n=5), whereas width and depth were calculated for all lesions: length, width, and depth were 24.6±9.7 (n=5), 15.6±4.6 (n=18), and 4.5±3.7 mm (n=18). We did not specifically intend to create PFA lesions in a linear fashion but some lesions were noted to be confluent. In these cases, only width was measured given the difficulty in estimating length (Figure S2). Two of 18 lesions were transmural over the RV, with RV wall thickness of 7.1 mm and 8.1 mm (one of these was at a site of lesion overlap).

Epicardial fat was seen during sectioning, in 3 of 18 lesions. The cross sections revealed similar depth of PFA lesions in the areas with or without epicardial fat, suggesting that a thin layer of fat (<4 mm) did not significantly attenuate PFA lesion depth (Figure 2B, 2D, and 2E). These three lesions’ sites had a maximum depth of 4.3±1.5 mm (range, 2.7–5.72 mm).

Coronary vessels were identified traversing the lesion path, in 5 of 18 cross sections, and appeared grossly spared with preserved lumens (Figure 2C, 2D, and 2F). Additionally, there had been no obvious ST-segment changes, wall motion abnormalities, or malignant VAs noted during epicardial PFA. Importantly, coronary angiography had not been performed during these PF applications—so subclinical coronary spasm cannot be excluded.

Endocardial lesions with this dose were applied only in the RV and revealed discrete lesions larger than those observed in experiment no. 1 with single PFA deliveries using the experimental PULSE2-1 dose. The size of the lesions was 19.8±2.7 mm, 15.0±2.3 mm, and 6.6±1.8 mm in length, width, and depth, respectively. Transmurality was observed in 5 of 10 (50%) lesions. There were no occurrences of VA in this evaluation as well (total applications delivered: 28).

### Experiment No. 3

During the initial procedure, a total of 18 clusters of PFA lesions were created: 10 in the RV and 8 in the LV. In the subsequent study 4 weeks later, 14 low voltage areas (healed scar) were identified, and PFA lesions were applied at multiple locations along the scar borders: 4 single and 22 repetitive applications (5 location with 2 applications, 1 location with 3 applications, 16 locations with 4 applications; Figure S1). No VA occurred during the initial procedure, but ventricular fibrillation (VF) occurred in 3 of 81 applications during ablation around the scar borders in the second procedure (all using the PULSE3 waveform optimized for atrial ablation rather than the PULSE3V waveform optimized for ventricular ablation).

**Figure 3.**
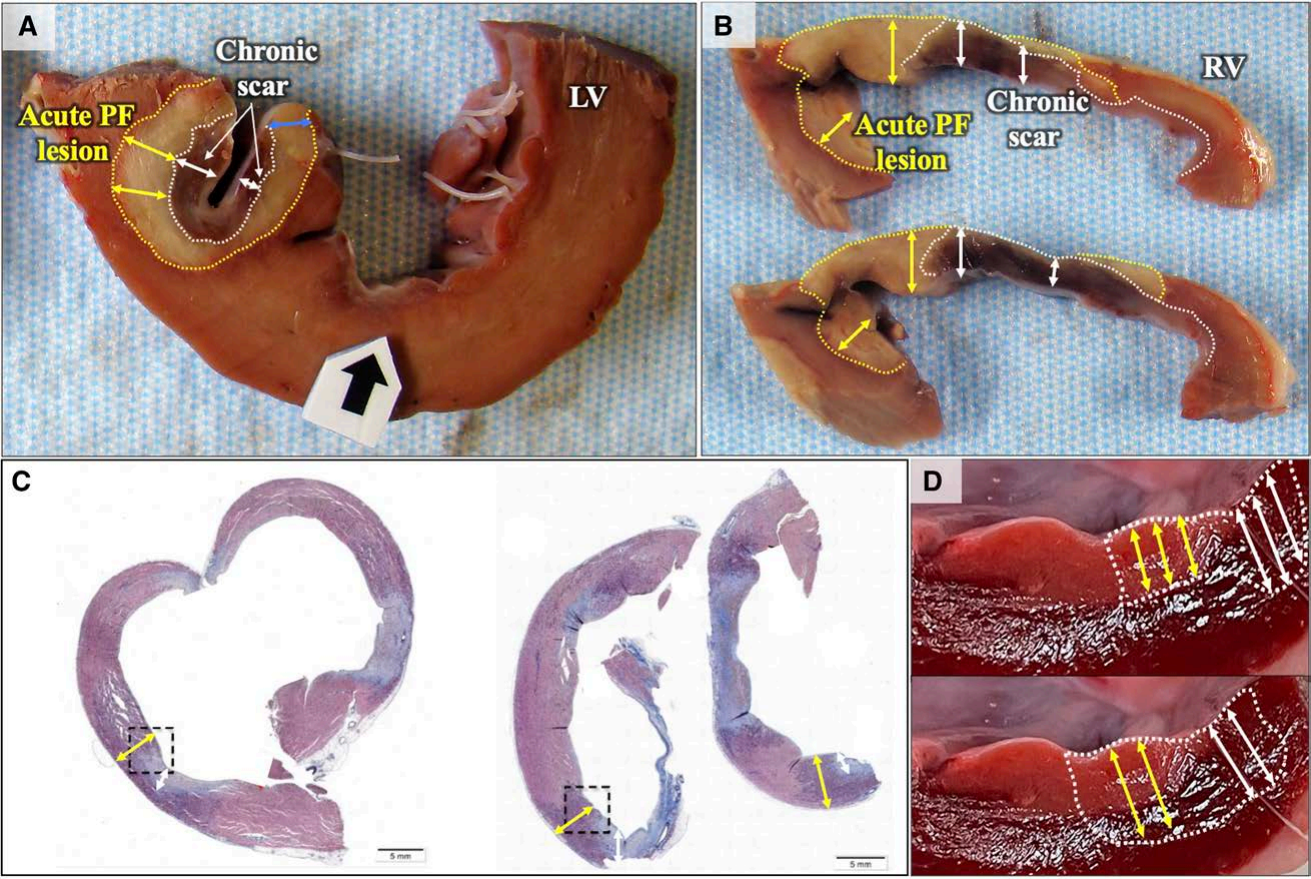
**Lesion-scar interaction. A**, Gross specimen demonstrating a nontransmural acute lesion placed upon a region of chronic scar in the left ventricle (LV). The white dotted line identifies the outer boundary of the chronic endocardial scar which appears dark brown/red (depth-double white arrows). Beyond this region, is a circumscribed pale region (yellow dotted line, yellow double arrows) that identified the acute pulsed field ablation (PFA) lesion that was placed over the chronic scar but is able to penetrate it. One can also appreciate that acute PFA (pale area) penetrates the chronic scar to the same depth, as is seen at the periphery of the chronic lesion (blue double arrow). **B**, Similar findings seen in a partially transmural right ventricle (RV) scar. **C**, Histology: Masson trichrome staining- Chronic fibrotic scar is stained light blue, whereas the acute lesion is stained light purple. Three lesions are shown in this part—all demonstrate extension of acute PFA (purple region) below and around the fibrotic scar. **D**, Experiment no. 3 Concept illustration: Pale pink region represents the outline of a chronic endocardial scar: **Upper**, Hypothesis: If PFA depth were to be impacted by presence of scar (pale pink region), then the acute lesion depth under endocardial scar would be diminished (yellow double arrow) compared to what is achieved in nearby healthy adjacent myocardium (white double arrows). **Lower**, Alternate hypothesis: If PFA depth were to be not impacted by scar- then lesion depth under the endocardial scar would not be diminished (yellow double arrow) compared to adjacent healthy myocardium (white double arrows).

The largest recorded voltage was 1.81±0.95 mV at baseline and reduced to 0.41±0.29 mV post-PFA (*P*<0.001). Of the 14 sites where PFA was delivered along the scar border, 7 acute lesions were identified to be adequately overlapping both scar and normal myocardium on gross necropsy. A total of 16 sections along the length of these lesions (10 RV and 6 LV) were analyzed. Acute lesions were easily identified as areas without triphenyl tetrazolium chloride uptake (pale) and were easily distinguished from the chronic scar (darker, fibrotic endocardial regions, with obvious volume loss compared to adjacent healthy regions; Figure 3A). The mean and maximum depth of the nontransmural chronic scar was noted to be 2.8±0.6 mm and 4.2±1.2 mm, respectively. The chronic scar lesions were homogenous in appearance with accompanying volume loss in comparison to the adjacent healthy myocardium.

Acute PFA lesions were identified extending beyond/deep to the scar in all examined sections (100%). The mean and maximum extent of acute PFA extending deep to the endocardial scar was 2.7±0.9 mm and 3.7±1.4 mm, respectively, with 10 out of 16 (62.5%) sections demonstrating complete transmural ablation (Figure 3A). In 8 of 16 sections, the acute PFA lesion was also seen extending into healthy myocardium immediately adjacent to endocardial scar. PFA was able to penetrate to a total depth of 4.8 (interquartile range, 4.5–5.4) mm in healthy myocardium versus 5.6 (interquartile range, 3.6–6.6) mm in adjacent regions of chronic scar (*P*=0.79; Figure 3A and 3B), suggesting that PFA lesions were noted to be similarly deep over scar and healthy myocardium. Select sections were submitted for histological assessment, which confirmed changes consistent with acute and chronic lesions in accordance with the gross appearance (Figure 3C).

### Histology

**Figure 4.**
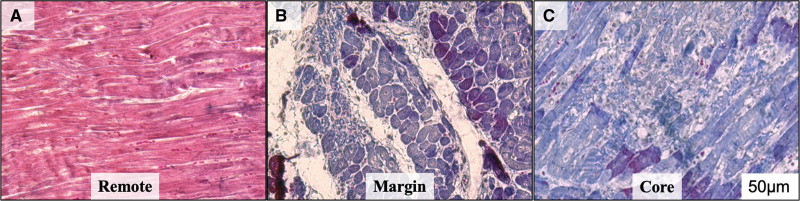
**Masson trichrome staining. A**, Healthy myocardium from a remote site in the left ventricle, (**B**) and (**C**) abnormal staining (blue) at the margin of the lesion and healthy myocardium (**B**) and from within the core of the lesion (**C**) suggesting myocardial damage.

**Figure 5.**
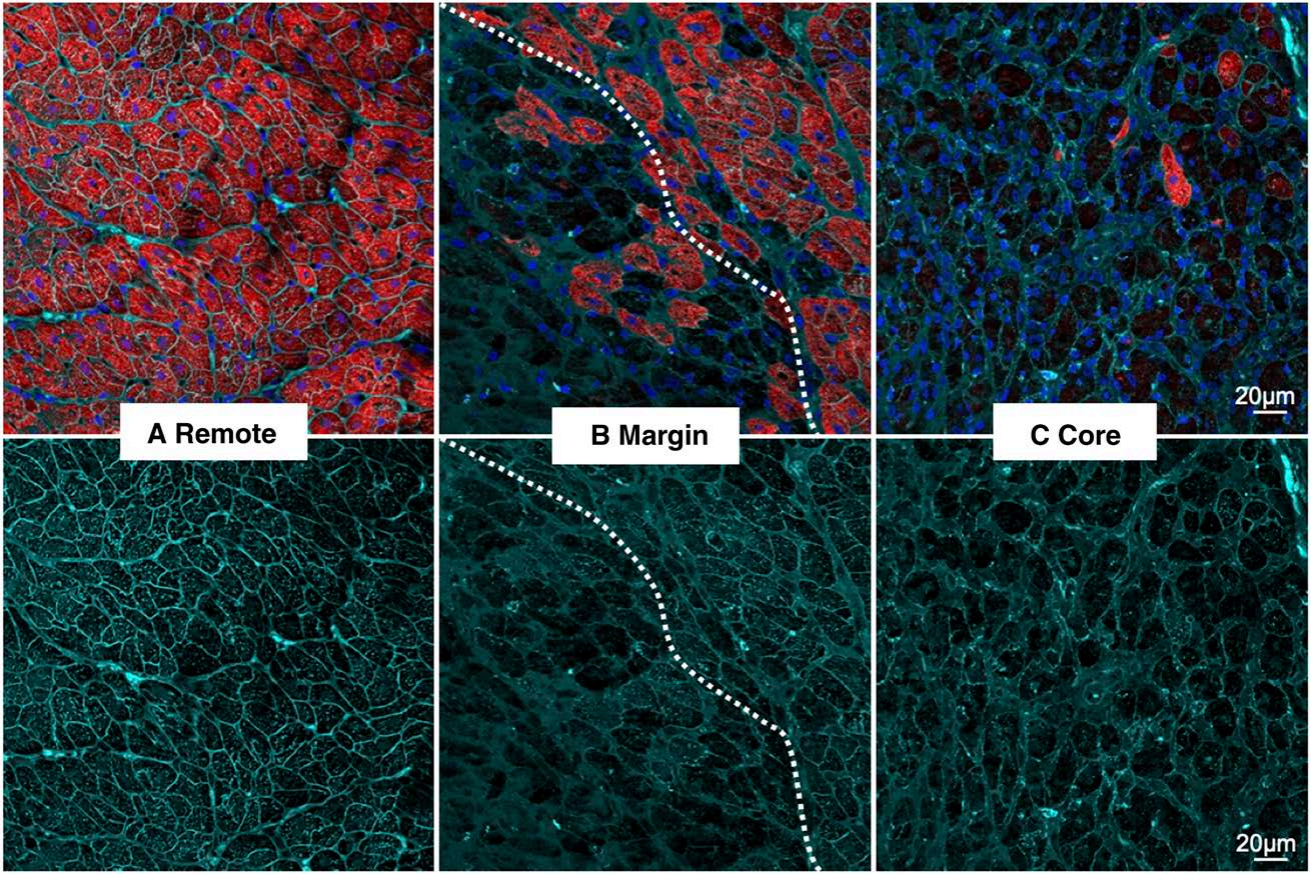
**Coimmunostaining of cTNI (cardiac troponin I) and WGA (wheat germ agglutinin). A**, remote healthy myocardium, (**B**) lesion margin and healthy myocardium (dotted line), and (**C**) lesion core. **A**, In remote areas, cardiomyocytes remain healthy, with compact and normal arrangement indicated by a defined cell membrane (WGA), (**B**) at the lesion margin, abrupt myocyte loss and smearing of cell membrane can be observed (dotted line identified the margin between live and dead myocytes). Loss of red CTNI staining represents damage to myocytes (**upper**)—note sharp margin of the lesion, (**C**) in the lesion core, almost all sarcomeric structures have disappeared and the myocytes exhibit distorted cell membrane. Sharply defined WGA-stained cell membranes surround the compact myocytes in remote regions (better appreciated in lower panel) but smears, diffusely into the expanded interstitial space in the lesion core. Color codes- cTNI: red, WGA: cyan, Nuclei: blue. Representative images are from 10 to 20 scans per area from 6 different sections. Scale bar, 20 μm.

**Figure 6.**
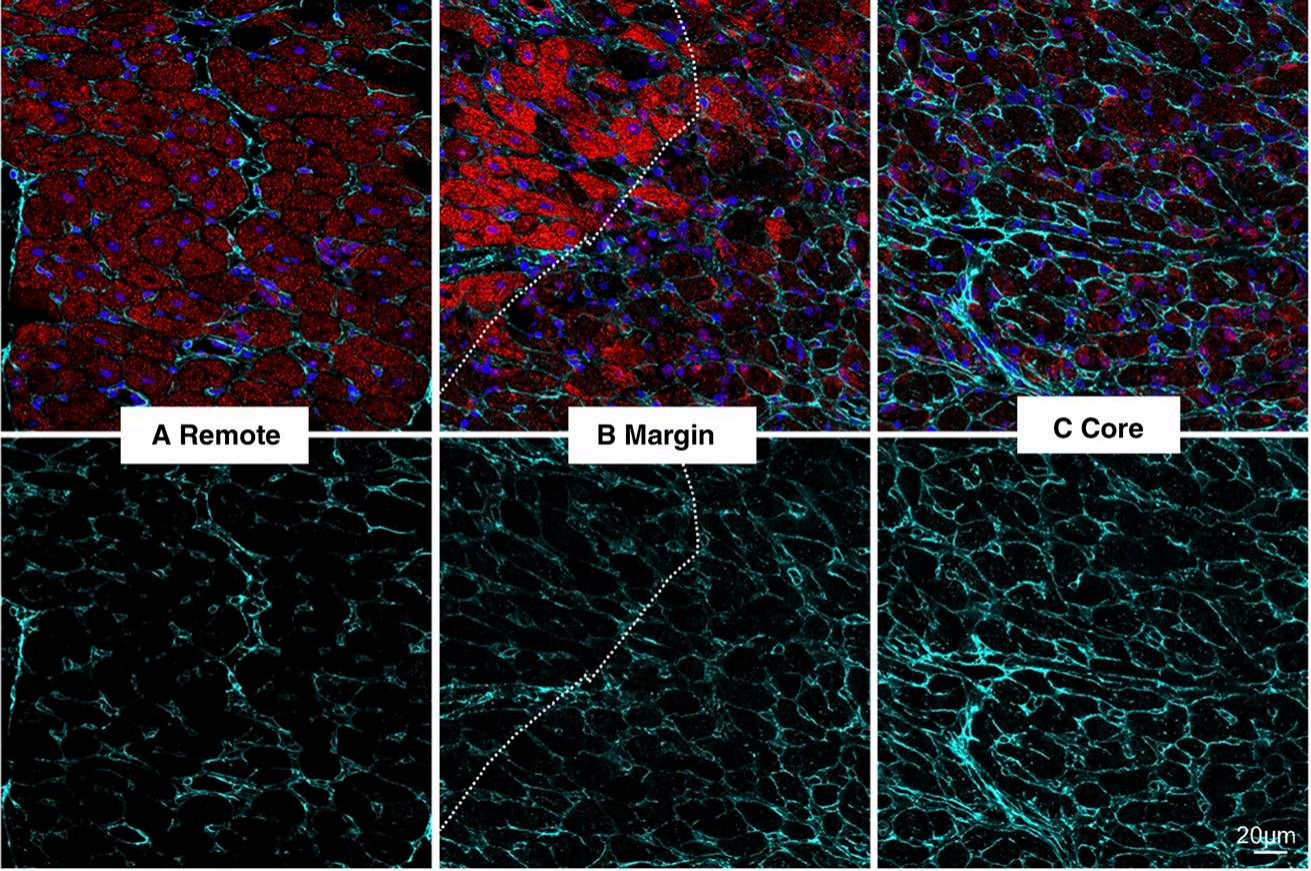
**Coimmunostaining of cTNI (cardiac troponin I) and Col IV (collagen IV). A**, Remote healthy myocardium, (**B**) lesion margin and healthy myocardium (dotted line), and (**C**) lesion core. **A**, In remote areas, baseline level of Col IV in uninjured myocardium. **B**, At the lesion margin, slightly increased Col IV is observed in the region with abrupt myocyte loss in comparison to adjacent region with surviving myocytes (**left** of the dotted line). Loss of red CTNT staining represents damage to myocytes (**upper**), (**C**) in the lesion core, significantly increased Col IV is observed where no surviving myocytes remains. Increased collagen deposition is seen in the lesion core. Color codes: cTNT: red, Col IV, cyan. Nuclei, blue. Representative images are from 10 to 20 scans per area from 6 different sections. Scale bar, 20 μm.

**Figure 7.**
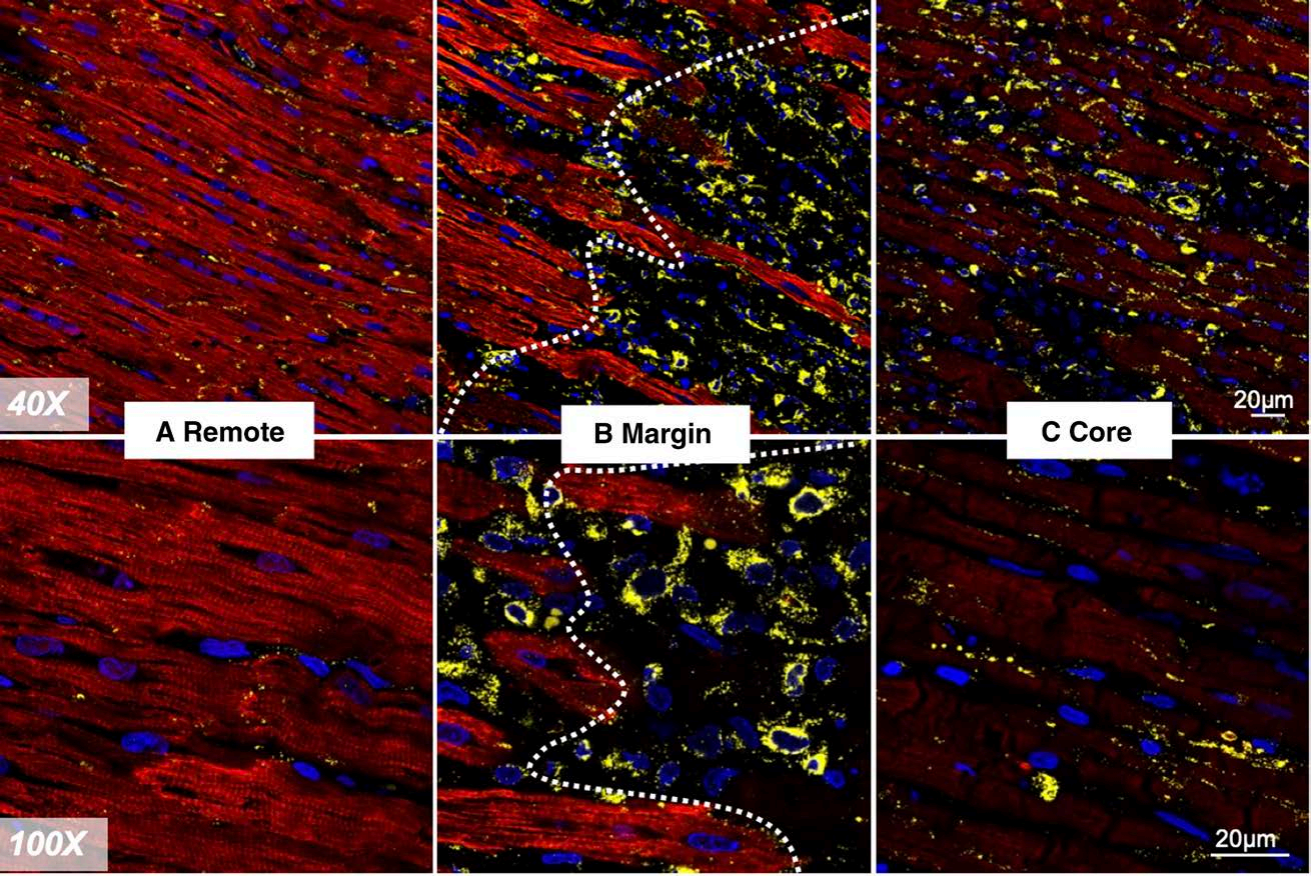
**Coimmunostaining of cTNI (cardiac troponin I) and CX43 (connexin-43). A**, remote healthy myocardium, (**B**) lesion margin and healthy myocardium (dotted line), and (**C**) lesion core. **A**, In the remote healthy myocardium, gap junction marker, CX43 is seen located at the end-end region between myocytes. **B**, At the margin, CX43 is seen accumulated into aggregates and have lost their normal cellular localization. Additionally, some aggregates are seen in a peri-nuclear distribution. Loss of red CTNI staining represents damage myocytes, (**C**) severe CX43 aggregates are seen in the lesion core. Color codes: cTNI, red. CX43, yellow. Nuclei, blue. Representative images are from 10 to 20 scans per area from 6 different sections. Scale bar, 20 μm.

From experiment no. 2, a subset of select lesions underwent hematoxylin and eosin and Masson Trichrome staining and immunohistochemical staining. Healthy cardiomyocytes and normal myofibril arrangement were noted at sampled remote (healthy and nonablated) sites. At lesion sites, sharp boundaries between healthy and ablated cardiomyocytes were identified as indicated by the loss of Masson trichrome staining (Figure 4). Deeper within the core of the lesion, increased collagen deposition was observed, accompanied by severe loss of cardiomyocytes. These findings correlated with the gross assessment of lesion margin and its core. Consistent with histology, immunohistochemistry demonstrated compact, healthy cardiomyocyte arrangement at remote sites with intact cell membranes (sharply defined WGA staining, Figure 5). Cardiomyocyte loss was observed beginning at the lesion margin with loss of sarcomeric marker cTnI (cardiac troponin I), accompanied by expanded extracellular space as indicated by smeared WGA expression and increased intermembrane distance. On the other hand, within the lesion core, there was severe loss of cardiomyocytes with no sharp WGA staining noted (Figure 5). Similarly, increased collagen deposition was seen beginning at the margin of the lesion and the lesion core (Figure 6). Furthermore, cardiomyocytes maintained normal tight junctions at remote sites with CX43 expression shown at the ends of adjacent healthy myocytes. However, abnormally increased CX43 aggregates were seen at the lesion margin and its core. Some myocytes remain striated at the lesion margin but most myocytes completely lost their sarcomeric structure within the core (Figure 7).

### ICE Imaging

Given the echogenicity and size of the lattice tip, the catheter tip was visualized for all PFA applications. During applications, the PF pulses saturated the EKG and intracardiac electrograms, so only mechanical effects of cardiac activation during the pulse delivery were identifiable.

We did not appreciate changes to the myocardium on ICE immediately after PFA, but regions of focal myocardial thickening with altered echogenicity at application sites were visible by the end of the study (<30 minutes postapplication, Figure 1A, Figure S3). During PF applications, microbubbles were observed; to differentiate their origin (saline irrigation versus PFA-related electrolysis), we delivered 5 separate PF applications in the center of separate chambers (left atrium, right atrium, and RV) with the catheter irrigation turned off: in all instances there were no microbubbles visible on ICE imaging, indicating that the previously observed microbubbles were due to saline irrigation rather than PFA delivery.

## Discussion

This in vivo evaluation offers several insights into the behavior of PFA delivered by the lattice-tip catheter into porcine ventricular myocardium:

1. Endocardial lesions are circular/oval in shape consistent with the expected geometry of the electrical field (Figure S4). Lesions are consistently detected, and are homogenous despite trabeculation with distinct margins, and are without hemorrhage.
2. Repetitive PF applications (2 through 4) progressively improve lesion depth. A mean depth of 6 mm, with length and width of ≈1.5 cm, is achievable.
3. Ventricular lesions can be visualized by ICE imaging acutely within minutes but not instantaneously. Myocardial mechanical activation occurs during pulse delivery and can be visualized on ICE.
4. Chronic lesions are associated with homogenous scar with accompanying volume loss.
5. Epicardial PFA is feasible and creates lesions with comparable dimensions, with preservation of surface contours.
6. Delivery of PFA optimized for ventricular tissue is feasible and results in comparable lesion dimensions to PFA optimized for atrial tissue.
7. Endocardial scarring up to 4 mm depth did not impair subsequent PFA from penetrating the myocardium beyond the scar. Thin layers of epicardial fat may not impair PFA delivery.

### Optimizing PFA Lesion Dimensions

Ventricular PFA using monophasic pulses have been previously described in several preclinical reports.^9-11^ Using custom catheters for epicardial ablation, large lesions could be created quickly and safely. In addition, they demonstrated sparing of epicardial coronary arteries and a dose-dependent increment in lesion size, raising interest in PFA’s potential role in clinical ventricular ablation. More recently, using modern biphasic waveforms delivered from a focal, large-tip multielectrode endocardial PFA catheter, we demonstrated the feasibility of large lesion creation by applying 2 to 4 applications; these were characterized by homogenous fibrous scar with discrete margins with sparing of nerves and large vessels.^6^

In our current report, we describe additional important observations using the lattice-tip catheter and its proprietary biphasic PFA waveform. We demonstrate that focal ventricular lesions can be successfully created using the lattice-tip PFA catheter (previously shown able to make transmural atrial lesions). This can be performed with excellent consistency: only one lesion was missing during pathological examination (possibly due to placement near the tricuspid valve), whereas lesions are often difficult to identify/create during preclinical studies of the smaller catheter tips that use conventional radiofrequency ablation. Lesion dimensions progressively increased with increased repetitions (2, 3, and 4 repeats), with the depth increasing from 4.2±1.0 mm (2× repetitions) to 6.1±2.1 mm (4× repetitions; *P*=0.003). This confirms and elaborates previous findings by both our group and recently by Yavin et al,^7^ who demonstrated improved depth by comparing 1 application against 4 repeat applications.

### PFA-Related Ventricular Activation

Activation of the ventricular myocardium occurs coincident to pulse delivery, and its occurrence can be easily missed as the electrocardiograms and intracardiac electrograms are obscured by the pulsing artifact. We think myocardial activation during PF delivery will likely prove to be a class effect across various PFA technologies, but additional studies are required to evaluate this hypothesis. This observation was noted during the exploratory evaluations performed in this report. PFA-related ventricular myocardial activation was identified by ICE imaging and lasted only the duration of the pulse delivery. The short duration of these pulse applications precludes any significant hemodynamic effects related to activation, although myocardial activation may itself terminate ongoing arrhythmias, such as ventricular tachycardia.

VF rarely occurred during PULSE2-1 and PULSE3 applications (1.0%, 3 of 305) and no VF occurred during PULSE3V applications (0%, 0 of 28). VF was observed following PFA around extensive scar (3.7%, 3 of 81) but was not observed at all following ablation in healthy myocardium (0%, 0 of 252). Those rare VF events may also have been influenced by the animal model used (swine are widely appreciated to be predisposed to VA during ventricular ablation, and none of the animals in this report received preprocedural oral amiodarone) and therefore should not be assumed to be conclusive in terms of risk of VF with PFA. One must also keep in mind that these are early, exploratory dosing experiments and that these observations are by no means reflective of the final delivery approaches that may be used clinically for ventricular PFA. However, the lack of any VF during PULSE3V delivery is reassuring, especially given the comparable ablation volumes seen with this approach but given the low number of applications with PULSE3V further study is essential. The observation of VA is also consistent with the expressed concern of VA with ungated PFA as mentioned in the cancer literature.^12-17^ VF, as an adverse event remains overall rare, in fact, Yavin et al^7^ did not report any VAs in their evaluation of PF using this catheter. These somewhat variable findings suggest that induction of VA while possible remains infrequent. Furthermore, the rare occurrence of induced VF can be readily addressed during a catheter ablation procedure, so the safety issue should be relatively minor. Further assessments of pulse modifications in different animal models (eg, canine/ovine) are ongoing.

### ICE Imaging

With respect to intraprocedural imaging of ventricular PFA, it is important to appreciate that the increased echogenicity was not noted immediately after the PFA pulse but required several minutes to develop. This finding has been observed with other PFA systems18; indeed, this phenomenon may also represent a class effect of PFA. Interestingly, PFA lesions are not usually visualized on necropsy when performed within minutes (Figure S2). These ultrasound findings deserve further study, as they may allow acute insight into PF lesion extent. Finally, myocardial activation during the PFA pulse is easily demonstrated on ICE, either by visualizing global ventricular contraction and the consequent valve opening/closing.

### Epicardial Ventricular PFA

The lattice tip was successfully maneuvered within the pericardial space to create high-density electroanatomical maps. The creation of epicardial lesions of similar dimensions is also important as this provides the basis for PFA to be applied for epicardial substrates. This builds on prior reports demonstrating the feasibility of epicardial PFA using surgical catheters to the epicardium.^9-11^ The known ability of PFA to spare the phrenic nerve and large epicardial vessels would be an added advantage but would require independent studies with this catheter. Indeed, our histology demonstrated spared epicardial nerves, arteries, and veins. However, we did not evaluate the potential for acute coronary spasm (though we did not observe wall motion abnormalities or ST-segment elevation), and therefore, this remains a complication that may be clinically relevant even if asymptomatic. The lack of saline/blood in the pericardial space did not impede PFA delivery, and lesions were easily created.

Although limited to 3 sections only, epicardial PF delivery to the underlying myocardium was not impeded by a thin layer of epicardial fat (<4 mm); in fact, when compared with adjacent areas with either less or no fat, the depth of PFA penetration was unchanged (Figure 2B and 2E). Gross examination of epicardial vessels also indicated that they were preserved with no evidence of disruption or hemorrhage along their course (Figure 2C)—their structural preservation was further confirmed by histological assessment (Figure 2D and 2F). This finding of epicardial fat not precluding PFA delivery is also demonstrated in an image from a recent report of epicardial PFA using a linear multielectrode catheter where the fibrotic PFA lesion can be seen below a region of epicardial fat.^19^ The role of epicardial fat and its impact on PFA delivery however needs more detailed and dedicated evaluation—especially in areas of fat with thicknesses >4 mm (since the fat thickness in each of the 3 sections were 3.05, 3.33, and 3.96 mm). Additionally, the role of contact, local impedance, and the presence of fluid (hypotonic or isotonic) in the pericardial space all are variables that require further study in addition to the effect on epicardial coronary arteries and the phrenic nerve.

### PFA on the Scar Tissue

To explore the effect of scar on PF lesion penetration (depth), we created a novel model of endocardium-only-homogenous scar by using the large footprint of the lattice tip to create islands of scar (without risking VA as is often seen with RF or ischemic models). The 4-week-old scar model does have limitations: (1) ischemic and nonischemic cardiomyopathies often have patchy, heterogeneous scar that is often significantly deeper, and (2) clinical scars are, of course, more chronic in duration and may have other components, such as calcification or intramural adipose tissue. However, this model allowed a very controlled and easy-to-identify investigation of the impact of endocardial scarring (Figure 3D). By examining PFA applications placed on scar boundary regions, we were able to compare the PFA lesion extent directly beneath the endocardial scar to the PFA lesion directly beneath the healthy myocardial region (without scar) located at the boundary. We were able to demonstrate that endocardial scar (to a maximum depth of 4.2 mm) does not impair PFA lesion formation, and this finding has important clinical implications.

### Histology and Immunohistochemistry of PFA

Additional observations can be drawn from histology/immunohistochemistry of the PFA lesions. Our findings suggest that cellular changes after PFA are clearly visible at the 24- to 48-hour time frame and confirm that gross changes with/without triphenyl tetrazolium chloride staining can also identify lesions accurately at this time point. As previously observed, the lesion-to-healthy myocardium border is distinct, with the transition occurring over a single layer of cells as shown clearly with the immunostains (as opposed to thermal lesions with a transition zone). In the core of the PF lesion, there was diffuse loss of sarcomeric proteins and expansion of the extracellular space with a mostly maintained cell architecture at this time point. Abnormal distribution of CX43 is in line with damage within the ablation lesion; importantly, these changes did not extend beyond the lesion border further confirming limitation of structural changes to the lesion zone. Further studies, that evaluate various doses as well as chronic timelines post-PFA are needed.

### Limitations

This preclinical evaluation was performed mostly in healthy swine, so one should be cautious in extrapolating these findings to healthy human or scarred myocardium. Individual experiments are of limited sample size given their exploratory nature and consequently impact the observations described. In particular, the PFA on scar model has limitations in its ability to offer relevant clinical insights into ischemic and nonischemic substrates, which have more chronic and heterogenous fibrosis.

### Conclusions

Focal PFA using a 9 mm lattice-tip catheter reliably creates wide endocardial or epicardial lesions that are homogenous with distinct margins. Repeating applications up to four times improves lesion dimensions. PFA pulses can be associated with local ventricular activation and potentially carries a risk of inducing VA, albeit infrequently; however, PFA delivery optimized for the ventricle may create comparable lesions without VA. Lesions can be visualized on intracardiac echocardiographic imaging acutely. Finally, 4-week-old endocardial scarring does not impair subsequent PFA lesions from penetrating the myocardium beneath the scar and this may have important clinical implications.

## Article Information

### Sources of Funding

This study was supported by a research grant from Affera, Inc.

### Disclosures

Dr Reddy and Koruth have received research grants from Affera, Inc. Drs Reddy and Koruth hold stock options in Affera, Inc. A comprehensive list of all financial disclosures (unrelated to this article) is included in the Supplemental Material. The other authors report no conflicts.

### Supplemental Material

Additional Disclosures

Figures S1–S4

## Supplementary Material
