# Supplementary material for: Pulsed Field Ablation of the Porcine Ventricle Using a Focal Lattice-Tip Catheter
Source: Circ Arrhythm Electrophysiol. 2022 Sep 8;15(9):e011120. doi: 10.1161/CIRCEP.122.011120 (PMC9794124; doi:10.1161/CIRCEP.122.011120)

# Supplemental Material

**<u>Table of Contents</u>:**

Page 2: Additional Disclosures
Page 3: Figure Legends
Page 4: Figure I
Page 5: Figure II
Page 6: Figure III
Page 7: Figure IV

**Additional Disclosures**

Vivek Reddy's disclosures with medical companies (unrelated to this manuscript) include: Abbott (Consultant), Ablacon (Consultant, Equity), Acutus Medical (Consultant, Equity), Apama Medical-Boston Scientific (Consultant, Equity), APN Health (Consultant, Equity), Aquaheart (Consultant, Equity), Atacor (Consultant, Equity), AtiAN (Consultant), Autonomix (Consultant, Equity), Axon Therapies (Consultant, Equity), Backbeat (Consultant, Equity), BioSig (Consultant, Equity), Biosense-Webster (Consultant), BioTel Heart (Consultant), Biotronik (Consultant), Boston Scientific (Consultant), Cardiac Implants (Consultant, Equity), CardiaCare (Consultant, Equity), Cardiofocus (Consultant), Cardionomic (Consultant), CardioNXT / AFTx (Consultant, Equity), Circa Scientific (Consultant, Equity), CoreMap (Consultant), CoRISMA (Consultant, Equity), Corvia Medical (Consultant, Equity), Dinova-Hangzhou Nuomao Medtech (Consultant, Equity), East End Medical (Consultant, Equity), EBR (Consultant), EPD-Philips (Consultant, Equity), EP Frontiers (Consultant, Equity), Epix Therapeutics (Consultant, Equity), EpiEP (Consultant, Equity), Eximo (Consultant, Equity), Farapulse-Boston Scientific (Consultant, Equity), Fire1 (Consultant), Gore & Associates (Consultant), HRT (Consultant, Equity), Impulse Dynamics (Consultant), Intershunt (Consultant, Equity), Javelin (Consultant, Equity), Kardium (Consultant, Equity), Keystone Heart (Consultant, Equity), LuxMed (Consultant, Equity), Manual Surgical Sciences (Equity), Medlumics (Consultant, Equity), Medtronic (Consultant), Middlepeak (Consultant, Equity), Neutrace (Consultant, Equity), Newpace (Equity), Nuvera-Biosense Webster (Consultant, Equity), Philips (Consultant), Pulse Biosciences (Consultant), Restore Medical (Consultant, Equity), Sirona Medical (Consultant, Equity), Surecor (Equity), Valcare (Consultant, Equity) and Vizaramed (Equity).

Srinivas Dukkipati's disclosures include: Biosense Webster (Research grant), Manual Surgical Sciences (Equity) and Farapulse which was acquired by Boston Scientific (Equity).

Jacob Koruth's disclosures with other medical companies include: Consultant – Abbott/Acutus/Kardium and Grant Support – Acutus/Cardiofocus/Kardium/LuxCath

# Supplemental Figure Legends

**Figure I. Electroanatomic endocardial bipolar voltage map of the right ventricle created using the lattice tip catheter. One can visualize the low voltage area of scar created by grouped PFA applications 4 weeks prior. New PFA lesions (green tags) are purposefully placed on the scar border (Experiment #3)**

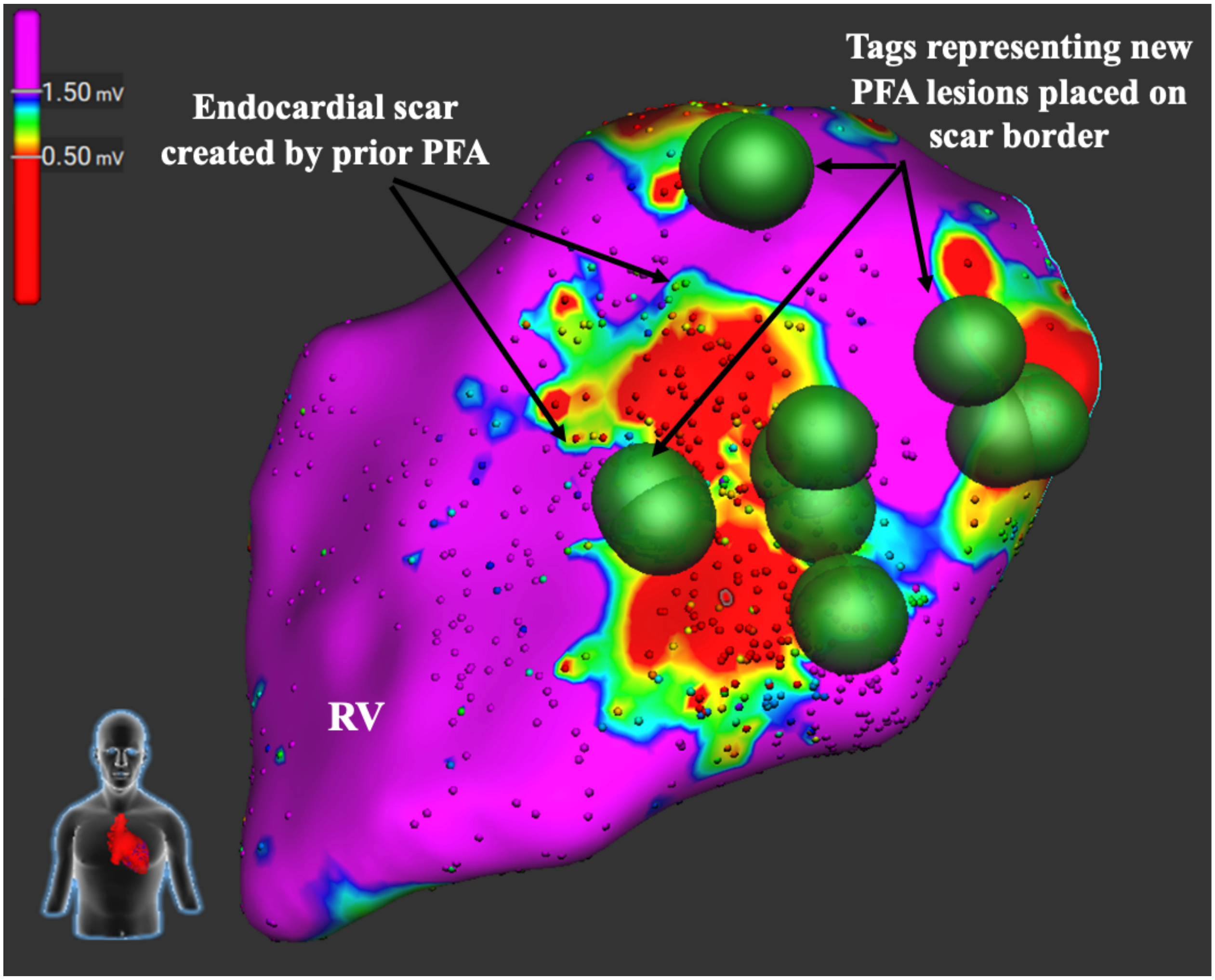

**Figure II. Example of lesion width measurement for confluent lesions.**

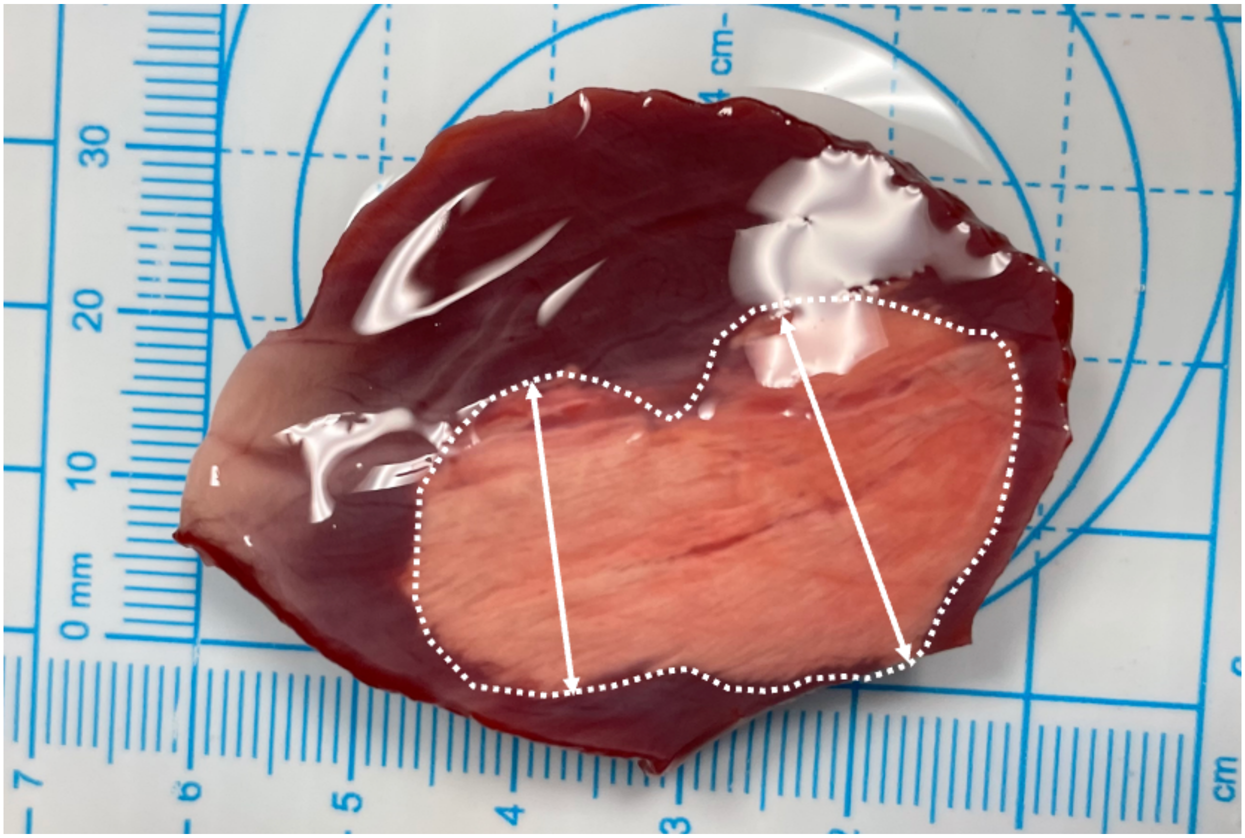

**Figure III. Intracardiac echocardiographic image demonstrating the right ventricular outflow tract free wall, interventricular septum and the left ventricle. The RV free wall demonstrates two adjacent regions of focal myocardial thickening at PFA applications (image captured within 30 minutes of PFA delivery).**

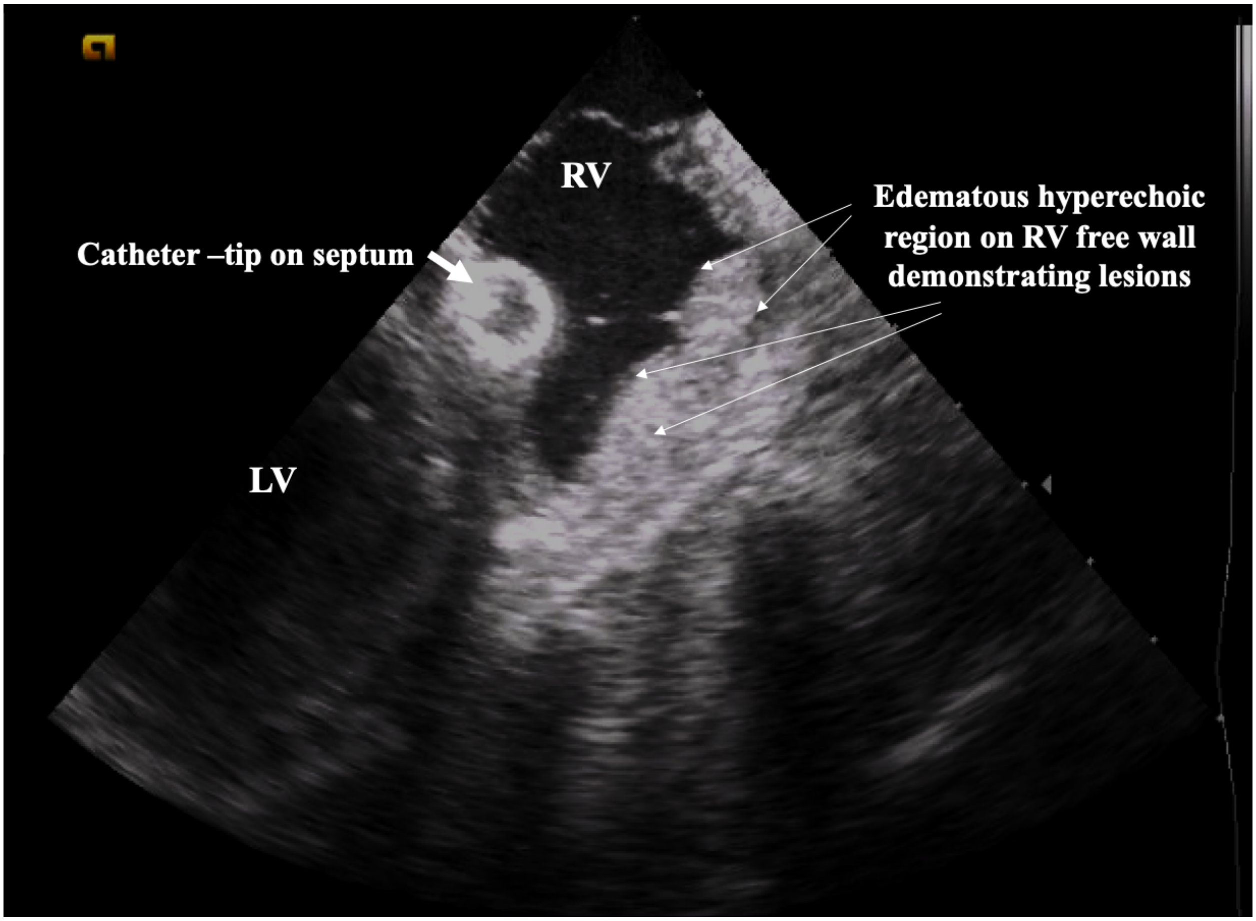

**Figure IV. This depicts the lattice-tip catheter (Sphere-9) and its E-Field isocontours.**

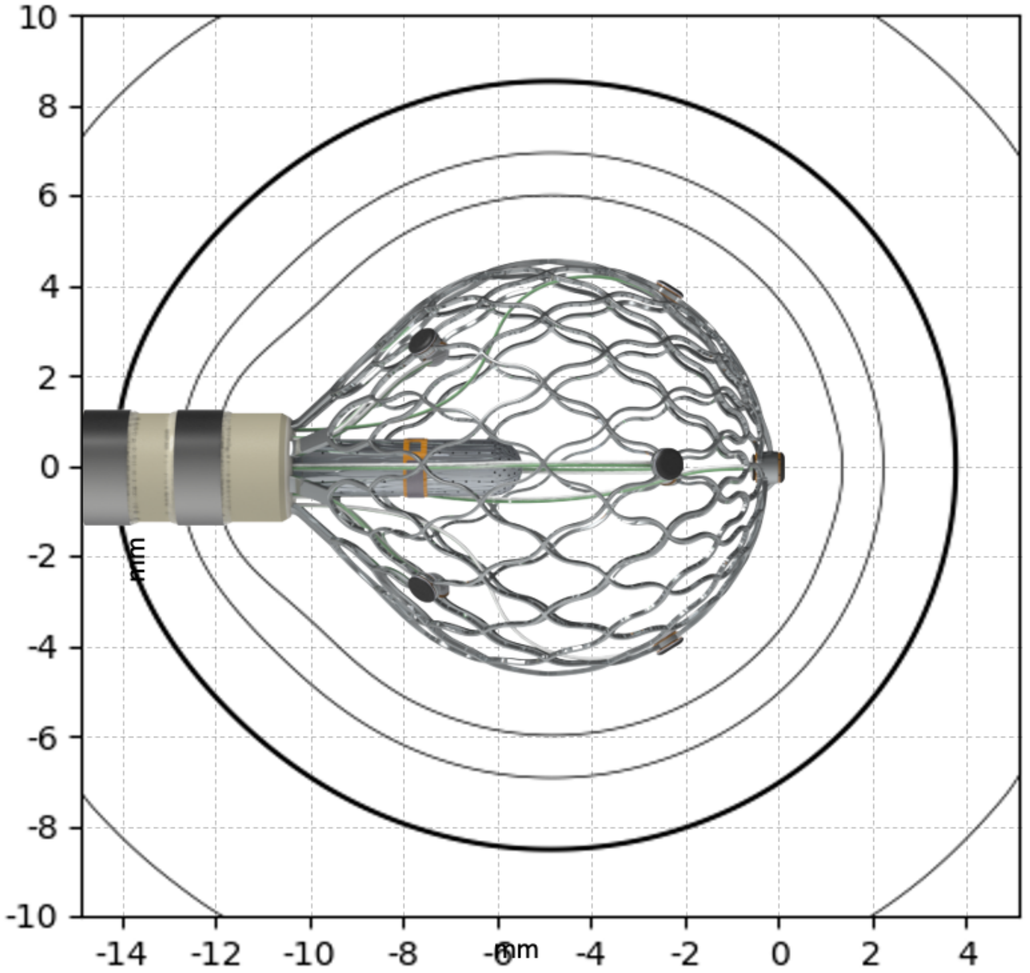

Supplement: Supplementary file 1 [file hae-15-e011120-s001.pdf]
